# Acute catatonia and psychosis in the context of seizure exacerbation in epilepsy: a case report

**DOI:** 10.3389/fpsyt.2026.1842802

**Published:** 2026-08-26

**Authors:** Maryam Alowais, Zainab Khan, Fares Salamah, Dania Khalil, Layla Murad, Sahar Marashi

**Affiliations:** 1 Graduate Medical Education, Mohammed Bin Rashid University of Medicine and Health Sciences, Dubai Health, Dubai, United Arab Emirates; 2 Psychiatry Department, Dubai Health, Dubai, United Arab Emirates

**Keywords:** antipsychotic, benzodiazepines, catatonia, consultation-liaison psychiatry, epilepsy, peri-ictal psychosis, seizure-associated psychosis, venlafaxine

## Abstract

**Background:**

The co-occurrence of seizure-associated psychosis and catatonia in epilepsy is diagnostically challenging, particularly when complex pre-existing psychiatric comorbidities complicate attribution. Few reports describe this triad in the context of an antidepressant switch.

**Case presentation:**

We report a 44-year-old woman with a 27-year history of epilepsy, maintained on carbamazepine and topiramate, and a longstanding psychiatric history of major depressive disorder, obsessive-compulsive symptoms, and chronic nihilistic delusions and intermittent hallucinations documented at baseline. One week before admission, her antidepressant was switched from clomipramine to venlafaxine 150 mg/day. Her caregiver reported increased seizure frequency, culminating in a breakthrough seizure 48 hours before presentation. She then developed acute mutism, food refusal, and unresponsiveness. On admission she met DSM-5-TR criteria for catatonia (mutism, stupor, posturing, waxy flexibility), which gradually resolved over the admission. Electroencephalography under sedation following benzodiazepine administration showed no epileptiform activity, though sensitivity for non-convulsive status epilepticus was limited. Electrocardiography demonstrated QTc prolongation (497 ms). Once verbal, the patient reported persecutory and nihilistic delusions, derealization, and auditory hallucinations. Management with escalating benzodiazepines and cautious antipsychotic uptitration (quetiapine XR, cariprazine) was associated with complete catatonic remission and psychotic symptom resolution over 29 days.

**Discussion:**

This case illustrates the diagnostic uncertainty inherent in attributing acute psychiatric features to seizure activity when complex pre-existing psychiatric histories exist. The absence of a documented lucid interval and the presence of chronic baseline psychotic symptoms preclude a confident diagnosis of postictal psychosis; interictal psychosis or primary psychiatric disorder cannot be excluded. The case highlights underrecognized pharmacokinetic interactions between carbamazepine (a potent CYP3A4 inducer) and second-generation antipsychotics, and cardiac safety considerations during antipsychotic uptitration with QTc prolongation.

**Conclusion:**

The working discharge diagnosis was catatonic disorder due to another medical condition (epilepsy), with psychotic features of indeterminate classification. Clinicians should resist anchoring on temporal associations between seizure activity and psychiatric symptoms. EEG findings must be interpreted in their pharmacological context; pharmacokinetic interactions should inform antipsychotic dose selection; and QTc prolongation warrants structured cardiac monitoring.

## Introduction

Epilepsy affects approximately 50 million people worldwide and carries an elevated risk of psychiatric comorbidity (1). Psychotic disorders occur in 6–10% of patients and are classified by their temporal relationship to seizures as ictal, postictal, or interictal (2, 3). Postictal psychosis classically requires a lucid interval between seizure cessation and psychotic symptom onset, and its boundaries with interictal psychosis, primary psychotic disorders, and delirium are frequently ambiguous (4). In patients with pre-existing chronic psychotic symptoms, this distinction is especially difficult to establish.

Catatonia — a neurobehavioral syndrome of mutism, stupor, posturing, waxy flexibility, and negativism — can arise in psychiatric, neurological, and medical conditions; its relationship with epilepsy remains undercharacterized (5, 6). It overlaps diagnostically with non-convulsive status epilepticus (NCSE) and postictal delirium, which may be difficult to differentiate even with EEG, particularly when EEG is performed under sedation or following benzodiazepines.

Psychotropic medication changes introduce additional risk. Several antidepressants, including venlafaxine, have been associated with dose-dependent seizure threshold lowering (7, 8). When psychiatric decompensation follows a medication change, attributing the presentation to pharmacological, epileptological, or primary psychiatric causes is challenging. Carbamazepine, a potent CYP3A4 inducer, may also reduce plasma concentrations of second-generation antipsychotics (9).

We present a case at the intersection of epilepsy, catatonia, and complex pre-existing psychiatric comorbidity, in the context of an antidepressant switch — not as a demonstration of postictal catatonia, but to illustrate the diagnostic uncertainty, pharmacological complexity, and safety challenges inherent in this scenario.

## Case presentation

### Patient information

A 44-year-old woman with a 27-year history of epilepsy and a longstanding psychiatric history presented with acute catatonic features following a breakthrough seizure temporally associated with an antidepressant switch.

### Neurological history

Epilepsy had been diagnosed at age 17, with seizures historically described as generalized tonic-clonic. Her regimen comprised carbamazepine 200 mg twice daily and topiramate 100 mg twice daily, with no missed doses before admission; seizures had been controlled prior to the antidepressant change. Brain MRI approximately seven years prior showed slight left mesial temporal lobe hyperintensity and was otherwise unremarkable. Prior EEG records were unavailable. Several features are inconsistent with this historical classification: the mesial temporal MRI abnormality, carbamazepine as a first-line agent typically used for focal epilepsy, and possible automatisms collectively suggest focal temporal lobe epilepsy with focal to bilateral tonic-clonic seizures (FBTCS) or focal impaired awareness seizures (FIAS). Definitive classification was not possible with available data. ASM serum levels were not measured, and no repeat neuroimaging was performed.

### Psychiatric history

She was first referred to psychiatry approximately 14 years prior for low mood, reporting a five-year history of depressive symptoms with death wishes. Major depressive disorder was diagnosed, and multiple medication trials followed over the subsequent decade. Obsessive-compulsive symptoms (intrusive religious doubts, compulsive ablutions and prayers) developed approximately two years later, with intermittent adherence difficulty. Electroconvulsive therapy had been administered approximately ten years prior.

Of particular diagnostic importance, her husband reported nihilistic delusions and intermittent hallucinations chronically present for at least two years prior to admission — not newly emergent. Whether these represented schizophrenia-spectrum disorder, mood disorder-related psychosis, or interictal psychosis of epilepsy was not established in the available records. No prior episodes of postictal psychosis or catatonia were documented. Baseline mental state findings immediately preceding the antidepressant switch were unavailable; this substantially limits differentiation of newly emergent from exacerbated pre-existing symptoms and is a significant limitation.

### Presenting concerns

Her husband reported worsening mood prompting a private psychiatrist to switch her antidepressant from clomipramine 125 mg/day to venlafaxine 150 mg/day approximately one week before presentation, after which he reported a marked increase in seizure frequency (approximately two daily) after previously stable control. This caregiver account is the sole evidence for seizure exacerbation; no objective documentation, neurological examination, or ASM serum levels were obtained. Two days before admission she had a breakthrough generalized tonic-clonic seizure that self-aborted without trauma. The following morning she became abruptly mute, refused food and medication, and was unresponsive to family. No clear lucid interval was documented between the seizure and behavioral change. In the emergency department she was wheelchair-dependent, followed only simple commands, and had poor eye contact with a fixed upward gaze.

### Mental state and catatonia assessment

On admission, the patient met DSM-5-TR criteria for catatonia, with four directly observed features: mutism (absent verbal response despite preserved hearing), stupor (no psychomotor activity, lying with knees flexed, minimally responsive), posturing (spontaneous maintenance of postures against gravity), and waxy flexibility (even resistance to repositioning, limbs maintaining placed positions). DSM-5-TR requires at least three of twelve features; this patient demonstrated four, fulfilling the criteria unambiguously (5). The Bush-Francis Catatonia Rating Scale (BFCRS) was not administered, an acknowledged limitation. The features persisted across the first several days, gradually improved with benzodiazepine therapy and antipsychotic augmentation, and resolved completely before discharge (**Table 1**) — a trajectory that further supports diagnostic validity. Consciousness was reduced with limited responsiveness; assessment of orientation, attention, and diurnal fluctuation was precluded by mutism. Temperature was normal throughout, and no neuroleptic malignant syndrome (NMS) features were identified.

**Table 1:** Timeline of clinical events and treatments.

Time point	Clinical and psychiatric events	Investigations and treatment
Pre-admission background		
~14 years prior	Initial referral; 5-year depressive history with death wishes; epilepsy on carbamazepine ~10 years.	Diagnosed with MDD; escitalopram commenced.
~12 years prior	Paranoid ideation; obsessive-compulsive symptoms; intermittent adherence.	Quetiapine added; multiple subsequent medication trials.
~7 years prior	Ongoing affective and psychotic symptoms despite treatment.	Brain MRI: slight left mesial temporal lobe hyperintensity. ECT trial.
Baseline (~2 years pre-admission)	Seizures controlled; chronic baseline nihilistic delusions and intermittent hallucinations (caregiver-reported, ≥2 years).	Clomipramine 125 mg/day; quetiapine XR; carbamazepine 200 mg BD; topiramate 100 mg BD.
Acute presentation		
1 week pre-admission	Worsening mood; antidepressant switched. Caregiver reported seizure increase to ~2/day.	Clomipramine 125 mg → venlafaxine 150 mg/day. ASM regimen unchanged. ASM serum levels not measured.
2 days pre-admission	Breakthrough generalized tonic-clonic seizure, self-aborted. Next morning: abrupt mutism, food/medication refusal, unresponsive. No documented lucid interval.	—
Day of admission (ED)	Mute; fixed upward gaze; wheelchair-dependent. Catatonic features (mutism, stupor, posturing) evident in ED. Afebrile, stable.	Levetiracetam 1,000 mg IV over 15 min. Diazepam 5 mg then lorazepam 1 mg — no response.
Inpatient admission (29 days)		
Day 1	Catatonia: four DSM-5-TR features — mutism, stupor, posturing, waxy flexibility (≥3 required; BFCRS not administered). Afebrile.	CT brain normal. ECG QTc 497 ms. K^+^ 3.3 mmol/L (low-normal; ref 3.3–4.8). Prolactin markedly elevated. EEG (sedated, post-BZD): no epileptiform activity. Lorazepam 1 mg BD; venlafaxine 150 mg OD; quetiapine XR 300 mg ON; ASMs continued.
Day 2	IV lorazepam challenge 2 mg total — no immediate response. Minimally verbal. Possible automatisms; repeat EEG not performed.	Lorazepam → 1 mg TDS. Quetiapine XR → 600 mg ON.
Day 3	Catatonia partially improved; once verbal, persistent psychotic features — persecutory and nihilistic delusions, derealization, auditory hallucinations (some self-harm content, no intent). Safety precautions maintained.	Cariprazine 1.5 mg BD initiated. NMS risk discussed; no NMS features.
Day 6	Catatonia persisting; bedbound. Ego-dystonic suicidal ideation — no intent or plan.	Lorazepam → 2 mg IV TDS. Enoxaparin 40 mg/day (VTE prophylaxis).
Day 9	Catatonia improving.	Lorazepam → 2 mg BD. Quetiapine XR → 300 mg ON. Cariprazine → 3 mg BD (6 mg/day).
Days 11–13	Continued improvement.	Lorazepam: IV → oral 2 mg BD.
Day 16	Catatonic features substantially improved; conversant; mobility restored.	Lorazepam stopped; clonazepam 1 mg BD. Quetiapine XR → 600 mg ON.
Discharge (Day 29)	All four catatonic features resolved. No psychosis, delusions, hallucinations, or suicidal ideation. Ambulating independently; self-care restored. Mild residual psychomotor slowing.	Discharge: quetiapine XR 600 mg ON; cariprazine 3 mg BD; venlafaxine 150 mg OD; clonazepam 1 mg BD; carbamazepine 200 mg BD; topiramate 100 mg BD. No follow-up data available.

ASM, antiseizure medication; BD, twice daily; BFCRS, Bush-Francis Catatonia Rating Scale; BZD, benzodiazepine; CT, computed tomography; DSM-5-TR, Diagnostic and Statistical Manual of Mental Disorders, 5th edition, text revision; ECG, electrocardiogram; ED, emergency department; EEG, electroencephalography; IV, intravenous; MDD, major depressive disorder; NCSE, non-convulsive status epilepticus; NMS, neuroleptic malignant syndrome; OD, once daily; ON, at night; TDS, three times daily; VTE, venous thromboembolism.

### Differential diagnosis

A systematic differential was constructed on admission and revisited throughout. Eight possibilities were considered:

1. Peri-ictal or postictal psychosis: supported by the temporal association with seizure; however, no lucid interval was documented, and baseline psychotic symptoms undermine confidence.
2. Interictal psychosis of epilepsy: equally plausible given the longstanding baseline psychotic symptoms.
3. NCSE or ictal catatonia: considered given the epilepsy history and possible automatisms; EEG carries important interpretive limitations (see below).
4. Postictal delirium: a key differential given reduced consciousness; assessment of orientation, attention, and diurnal fluctuation was precluded by mutism, limiting differentiation.
5. Schizophrenia or major depressive disorder with psychotic features: plausible given the decade-long psychiatric history; may account for the psychotic features independently of seizures.
6. Medication-induced seizure threshold lowering: venlafaxine’s propensity to lower the seizure threshold was considered as a potential pharmacological contributor (7, 8).
7. Metabolic or toxic etiology: addressed systematically by laboratory investigations.
8. Autoimmune encephalitis: not formally excluded; anti-NMDA receptor antibody testing, CSF examination, and toxicology were not performed — an acknowledged limitation (10).

### Investigations

Admission bloods showed potassium at the lower limit of normal (K^+^ 3.3 mmol/L; reference 3.3–4.8), mildly elevated inflammatory markers, mild transaminitis, mild microcytic anemia, and markedly elevated prolactin (consistent with antipsychotic exposure and/or recent seizure). Thyroid function, glucose, and acid-base status were unremarkable; CT brain showed no acute pathology. ECG demonstrated QTc prolongation at 497 ms, warranting careful cardiac monitoring during antipsychotic uptitration (11). EEG, performed under sedation following prior lorazepam, showed no epileptiform discharges; generalized beta activity was attributed to benzodiazepine effect. This requires caveats: benzodiazepines suppress ictal activity and may produce a falsely reassuring EEG, sedation reduces signal quality, and a single recording cannot exclude NCSE across the full episode (12). Repeat, prolonged, or video-EEG was not performed despite possible automatisms — a substantive diagnostic gap.

### Therapeutic interventions and clinical course

In the emergency department, levetiracetam 1,000 mg IV (in 0.9% sodium chloride 100 mL over 15 minutes) was given for acute seizure management. Diazepam 5 mg and lorazepam 1 mg were given sequentially without discernible response.

She was commenced on scheduled lorazepam 1 mg twice daily, venlafaxine 150 mg once daily, quetiapine XR 300 mg nightly, and her pre-existing ASM regimen. The following day, an IV lorazepam challenge (1 mg, repeated once; 2 mg total) was administered. This falls at the lower end of the standard 2–4 mg range (5, 13); the absence of immediate response does not refute the diagnosis. She became minimally verbal when addressed by family, with intermittent non-purposeful behavior suggestive of automatisms; repeat EEG was not obtained.

Once verbal, she reported persecutory delusions, nihilistic delusions (uncertainty whether she was alive), derealization, and distressing intrusive images. Auditory hallucinations were thought-like, with intermittent self-harm content; she denied intent, had no access to sharp objects, and safety precautions were maintained throughout.

Cariprazine was introduced on day three at 1.5 mg twice daily for persistent psychotic features, and uptitrated to 3 mg twice daily (6 mg/day total) by day nine. Antipsychotic use in catatonia — where caution is advised given NMS risk — was justified by the severity and persistence of psychosis causing impairment beyond the catatonia; no NMS features emerged. Two pharmacokinetic considerations applied: carbamazepine is a potent CYP3A4 inducer that may reduce plasma concentrations of CYP3A4-metabolized antipsychotics, including quetiapine, and for cariprazine specifically co-administration with strong CYP3A4 inducers is generally not recommended (9), an important safety and interpretive limitation; and venlafaxine, quetiapine, and cariprazine each carry propensity to lower the seizure threshold, with continuation based on the judgment that concurrent antiepileptic therapy provided adequate protection. Twice-daily dosing was used for incremental titration, though once-daily dosing is conventional given the drug’s extended half-life (14).

By day six, persistent immobility necessitated further lorazepam escalation and prophylactic anticoagulation. She disclosed ego-dystonic suicidal ideation without intent or plan; safety precautions were reinforced. Gradual, sustained improvement in catatonic features, mobility, and psychiatric symptoms followed over three weeks, with sequential benzodiazepine taper and antipsychotic optimization (**Table 1**).

Regarding cardiac safety, admission ECG showed QTc prolongation (497 ms) and serum potassium was at the lower limit of the local reference range (3.3 mmol/L; reference 3.3–4.8). Although supplementation was not undertaken, electrolyte optimization and serial ECG monitoring would have been preferable given the QTc prolongation and ongoing antipsychotic uptitration. The risk–benefit reasoning favoring treatment over arrhythmia risk was not formally documented, and the absence of serial cardiac monitoring is a safety limitation.

### Follow-up and outcomes

By discharge on day 29, she was conversant and ambulating independently, with restored oral intake and self-care. All four catatonic features had fully resolved on serial examination, with no ongoing hallucinations, delusions, or suicidal ideation. Residual psychomotor slowing was present but substantially improved. No adverse events (falls, aspiration, thromboembolism, or NMS) occurred. The working discharge diagnosis was catatonic disorder due to another medical condition (epilepsy) per DSM-5-TR, the psychotic features being of indeterminate classification. Post-discharge follow-up data were not available within our facility, precluding assessment of sustained remission, seizure recurrence, and long-term pharmacological safety. This represents a substantive limitation of the report.

## Discussion

This case illustrates the diagnostic and therapeutic complexity of the epilepsy–psychiatry interface across four domains: diagnostic uncertainty; epilepsy classification; EEG interpretation; and pharmacological safety.

### Diagnostic uncertainty: why postictal psychosis cannot be assumed

The central challenge is the tension between the acute temporal association with seizure activity and the longstanding psychiatric history. Postictal psychosis classically requires a lucid interval — typically 12 to 72 hours — between seizure cessation and psychosis onset (4). Neither this nor a clear return to baseline was documented, and the chronic baseline psychotic symptoms make it impossible to determine which features were new-onset versus exacerbated. Prevalence estimates of 6–10% in refractory epilepsy (2–4, 16) derive from populations in which primary psychiatric disorders were excluded — an exclusion that cannot be assumed here.

The presentation may equally represent interictal psychosis or a primary psychotic disorder coincidentally associated with the seizure. Clinicians must resist anchoring on the most dramatic temporal association; the discharge diagnosis should be understood as a working formulation rather than a confirmed causal attribution.

### Epilepsy classification and its implications

Several features are inconsistent with the historical generalized tonic-clonic classification: the left mesial temporal MRI abnormality, carbamazepine (a first-line focal-epilepsy agent), and possible automatisms collectively suggest focal temporal lobe epilepsy with FBTCS or FIAS (15). Temporal lobe epilepsy carries a higher risk of interictal psychosis than other syndromes (2, 3), further supporting it as a plausible alternative. Updated EEG with ictal recording and contemporaneous MRI are warranted.

### EEG interpretation in catatonia

Catatonia in epilepsy can mimic, or be mimicked by, postictal confusion and NCSE (5, 6). The EEG here — under sedation following lorazepam — requires careful interpretation: benzodiazepines suppress epileptiform discharges, so a normal EEG excludes only ictal activity at the moment of recording in that pharmacological state (12). This is particularly consequential given the observed automatisms, which in known epilepsy with a fluctuating mental state indicate prolonged or continuous EEG monitoring. Catatonia and NCSE can coexist; their distinction has treatment implications not fully resolvable here.

### Pharmacological safety: drug interactions and cardiac risk

Three pharmacological challenges merit discussion. First, antipsychotic use in catatonia requires justification given NMS risk (5, 13); here the severity of persistent psychosis outweighed the risk, and no NMS features emerged. Second, carbamazepine is a potent CYP3A4 inducer that may reduce plasma concentrations of CYP3A4-metabolized antipsychotics; for cariprazine, co-administration with strong inducers is generally not recommended (9), and should be prospectively documented. Third, QTc prolongation (497 ms) during antipsychotic uptitration requires a documented risk–benefit assessment and serial ECG monitoring (11); the failure to repeat ECG represents a safety gap that should inform future practice.

### Strengths and limitations

This report describes a complex presentation at the catatonia–epilepsy interface with explicit acknowledgment of uncertainty. Eight limitations constrain the conclusions: (1) seizure exacerbation was based solely on caregiver report, without objective documentation or ASM serum levels; (2) no lucid interval was documented, precluding confident postictal psychosis classification; (3) EEG was performed under sedation after benzodiazepines without repeat recording, despite automatisms; (4) the BFCRS was not administered, though the diagnosis rested on unambiguous fulfillment of four DSM-5-TR features and their resolution with treatment; (5) toxicology, CSF examination, and autoimmune encephalitis evaluation were not performed; (6) baseline psychiatric documentation before the switch was unavailable, limiting differentiation of new versus exacerbated symptoms; (7) serial ECG monitoring was not undertaken; and (8) post-discharge follow-up was unavailable. These reflect the real-world constraints of acute inpatient practice and are reported transparently.

## Conclusion

In a 44-year-old woman with epilepsy and complex psychiatric comorbidities, an antidepressant switch was temporally associated with caregiver-reported seizure exacerbation, a breakthrough seizure, and acute catatonia meeting DSM-5-TR criteria (mutism, stupor, posturing, waxy flexibility). The working discharge diagnosis was catatonic disorder due to another medical condition (epilepsy) per DSM-5-TR, the psychotic features being of indeterminate classification. Postictal psychosis was specifically not diagnosed, given the chronic baseline psychotic symptoms, absent lucid interval, and plausibility of interictal psychosis or primary psychiatric disorder. Management with escalating benzodiazepines and cautious antipsychotic augmentation achieved full catatonic remission and functional recovery over 29 days. Four lessons emerge: (i) diagnostic humility is essential when attributing psychiatric features to seizures in complex histories; (ii) a single post-benzodiazepine sedated EEG is insufficient to exclude NCSE when automatisms are present; (iii) carbamazepine’s CYP3A4 induction should inform antipsychotic selection; and (iv) QTc prolongation during antipsychotic uptitration demands structured cardiac monitoring (**Table 2**).

**Table 2:** Key learning points.

Learning point	Key concept	Clinical implication
Diagnostic humility	Postictal psychosis cannot be confidently diagnosed when chronic psychotic symptoms predate the presentation.	Use temporal rather than causal language; consider interictal psychosis and primary psychotic disorders as alternatives.
EEG interpretation	A single sedated post-benzodiazepine EEG cannot exclude NCSE, particularly when automatisms are observed.	Pursue prolonged or repeat EEG when ictal phenomena cannot be excluded; interpret negative EEGs in context.
CYP3A4 interactions	Carbamazepine is a potent CYP3A4 inducer that may substantially reduce plasma concentrations of CYP3A4-metabolized antipsychotics; co-administration with cariprazine is generally not recommended.	Account for this interaction when selecting antipsychotics in patients on carbamazepine; for quetiapine, monitor response and consider therapeutic drug monitoring where available.
Antipsychotics in catatonia	Cautious use is advised due to NMS risk; antipsychotics may be justified when psychosis persists despite adequate benzodiazepines.	Document the risk–benefit rationale; monitor for NMS and catatonic worsening; consider ECT if pharmacotherapy fails.
Cardiac safety	QTc prolongation during antipsychotic uptitration requires structured cardiac monitoring.	Obtain serial ECGs during titration; document reasoning; arrange post-discharge cardiac follow-up.
Catatonia rigor	Document DSM-5-TR criteria feature-by-feature (here four features met). Structured scales (e.g., BFCRS) standardize severity.	Routinely apply BFCRS or equivalent; follow established lorazepam challenge protocols (2–4 mg).
Multidisciplinary care	Catatonia at the epilepsy–psychiatry interface requires coordinated psychiatry, neurology, and pharmacy input.	Establish early multidisciplinary involvement; plan cross-specialty post-discharge follow-up.

## Patient perspective

The patient described the acute episode as deeply distressing, with intrusive, ego-dystonic suicidal thoughts she did not wish to act upon, expressing a clear desire to live. She experienced the gradual return of speech and mobility as a profound relief and expressed gratitude for her care.

## Data Availability

The original contributions presented in the study are included in the article/supplementary material. Further inquiries can be directed to the corresponding author.
